# Stem cell function is conserved during short-term storage of cultured epidermal cell sheets at 12°C

**DOI:** 10.1371/journal.pone.0232270

**Published:** 2020-05-20

**Authors:** Håkon Ringstad, Sjur Reppe, Tine Hiorth Schøyen, Kim Alexander Tønseth, Tor Paaske Utheim, Catherine Joan Jackson

**Affiliations:** 1 Department of Medical Biochemistry, Oslo University Hospital, Oslo, Norway; 2 Faculty of Medicine, Institute of Clinical Medicine, University of Oslo, Oslo, Norway; 3 Department of Plastic and Reconstructive Surgery, Oslo University Hospital, Oslo, Norway; 4 Lovisenberg Diaconal Hospital, Unger-Vetlesen Institute, Oslo, Norway; 5 Department of Clinical Medicine, Faculty of Health Sciences, The Arctic University of Norway, Tromsø, Norway; 6 Department of Ophthalmology, Oslo University Hospital, Oslo, Norway; 7 Department of Ophthalmology, Sørlandet Hospital Arendal, Arendal, Norway; 8 National Centre for Optics, Vision and Eye Care, University of South-Eastern Norway, Kongsberg, Norway; 9 Department of Ophthalmology, Vestre Viken Hospital Trust, Drammen, Norway; 10 Department of Ophthalmology, Stavanger University Hospital, Stavanger, Norway; 11 Department of Clinical Medicine, Faculty of Medicine, University of Bergen, Bergen, Norway; 12 Department of Dentistry, Institute of Oral Biology, University of Oslo, Oslo, Norway; Newcastle University, UNITED KINGDOM

## Abstract

Transplantation of cultured epidermal cell sheets (CES) can be life-saving for patients with large area burns. CES have also been successfully used to regenerate eye and urethral epithelia in animal models. Short-term storage aims to extend the transplantation window, offers flexibility in timing surgery and allows testing of CES quality, phenotype and sterility. This study investigated extended CES storage and explored the effect of additional re-incubation recovery time following storage. The proliferative quality of stored confluent versus pre-confluent CES was also investigated using functional testing. CES were stored at 12°C and results compared to non-stored control CES. Investigation of timepoints during 15 days storage revealed that viability began to deteriorate by day 11 and was associated with increased lactate in the storage medium. The percentage of apoptotic cells also significantly increased by day 11. Flow cytometry analysis of integrin β1 expression and cell size indicated best retention of stem cells at 7 days of storage. Functional testing of pre-confluent and confluent cells following 7 days storage showed that pre-confluent cells responded well to 1-day re-incubation after storage; they became highly prolific, increasing in number by ~67%. Conversely, proliferation in stored confluent cells declined by ~50% with 1-day re-incubation. Pre-confluent stored CES also had far superior stem cell colony forming efficiency (CFE) performance compared to the confluent group. Re-incubation improved CFE in both groups, but the pre-confluent group again out-performed the confluent group with significantly more colonies. In conclusion, a maximum storage period of 7 days is recommended. Use of pre-confluent cells and one day recovery incubation greatly improves viability, colony-forming ability and proliferation of cells stored for 7 days at 12°C. Thus, these recommendations should be considered under culture and storage of high-quality CES for clinical use.

## Introduction

Cultured epidermal cell sheets (CES) have been used as a life-saving treatment for patients with severe large area burns since 1984 [1]. They have since been applied to treat skin ulcers and recent studies indicate that CES also have potential for use in regenerative medicine applications such as urethral reconstruction [2] and corneal regeneration in limbal stem cell deficiency [3]. Preparation of CES requires specialist knowledge and cell culture laboratories. We anticipate that increased demand for cell-based treatments using CES will likely lead to stricter regulatory standards in cell culture laboratories, increased costs and centralization of cell culture facilities [4]. Short-term storage and transportation of CES could be key to meeting heightened clinical demand and provide worldwide access to cell-based regenerative medicine treatments [5].

Several technical and clinical challenges need to be overcome for successful transplantation of CES. Ideally, CES should be transplanted as soon as possible to ensure graft integration at the wound site [6]. Optimal timing of transplantation to accommodate patient needs may be difficult to coordinate with graft preparation as it takes several weeks to culture CES from biopsy to finished graft [6, 7]. Short-term storage aims to preserve the proliferative potential and stem cell function of CES and extend the transplantation window providing flexibility. Storage also provides an option for multiple rounds of surgery, which is often required in patients with large area burns. Furthermore, the storage period provides a window for testing CES quality, phenotype and sterility.

We have established that storage of CES at the optimum temperature of 12°C can extend their useful period for at least 7 days [8–10]. The present study aimed to investigate important indicators of CES quality in the interval between 7–15 days to establish the optimal storage period. We also wished to improve the storage method by analyzing the effect of cell confluency at the time of storage and addition of a recovery period (incubator settings 37°C and 5% CO_2_) following storage. It has been shown that CES can be cryopreserved at 70–80% confluence at -80°C with a one-day re-incubation recovery period [11]. An advantage of pre-confluent cell cultures (covering up to 80% of the culture dish) is that they have high proliferative capacity compared to 100% confluent cell sheets as confluence is a signal for cell differentiation [12]. We hypothesized that re-incubating CES following storage at 12°C would allow the cells to normalize cellular function. If proliferative capacity can be maintained during storage this could result in better graft integration and a higher rate of proliferation following transplantation to the patient.

## Materials and methods

### Supplies

Proprietary cell culture medium CnT-Prime was purchased from Cellntec Advanced Cell Systems AG (Bern). Trypsin-ethylenediaminetetraacetic acid (EDTA), soybean trypsin inhibitor, sodium bicarbonate, goat serum, sodium azide, Tween-20, 4-(2-hydroxyethyl)-1-piperazineethanesulfonic acid (HEPES), Triton X-100, gentamicin, bovine serum albumin (BSA), fetal bovine serum (FBS), penicillin-streptomycin, human recombinant insulin, hydrocortisone, adenine, 3,3,5-triiodo-L-thyronine (T3), dispase II, and trypan blue were purchased from Sigma Aldrich (St Louis, MO). Cholera toxin was purchased from Enzo Life Sciences (Farmingdale, USA). Epidermal growth factor, Gibco low-glucose Dulbecco’s modified Eagle’s medium (DMEM), Gibco 1:1 DMEM/F12 nutrient mix, minimal essential medium (MEM), phosphate buffered saline (PBS), Hank’s balanced salt solution, routine plastics, pipettes and Nunclon Δ surface multi-dishes were purchased from Thermo Fisher Scientific (Life Technologies) (Waltham, MA).

### Cell isolation

After obtaining informed written consent and approval from the local ethics committee (Regional Ethical Committee for Medicine and Health South-east Norway reference: 2013/815/REK South-east C) in accordance with the Declaration of Helsinki, epidermal keratinocytes were isolated from skin from a living donor. The donor was a 21-year-old female undergoing breast reduction surgery who gave informed written consent for use of the tissue. Cell isolation and all experiments were carried out in accordance with relevant guidelines and regulations set out by the local ethics committee. Primary epidermal keratinocytes were isolated from donor dermis and cultured according to the protocol previously described [10]. Briefly, adipose tissue was removed, leaving the epidermis and a thin dermis. 2 cm × 0.3 cm pieces were left overnight at 4°C with 1 ml of 1:1 dispase II + CnT-Prime medium with 100 μg/ml penicillin and streptomycin. Epidermis was separated from dermis and incubated with trypsin (0.025% + 0.01% EDTA) for 10 minutes at 37°C. Trypsin was neutralized with soybean trypsin inhibitor and primary cells were seeded at 20000 cells/cm^2^ in serum-free CnT-Prime culture medium in 80 cm^2^ flasks coated with 1 μg/cm^2^ collagen IV (BD Biosciences, New Jersey, USA).

### Cell culture

**Fig 1:**
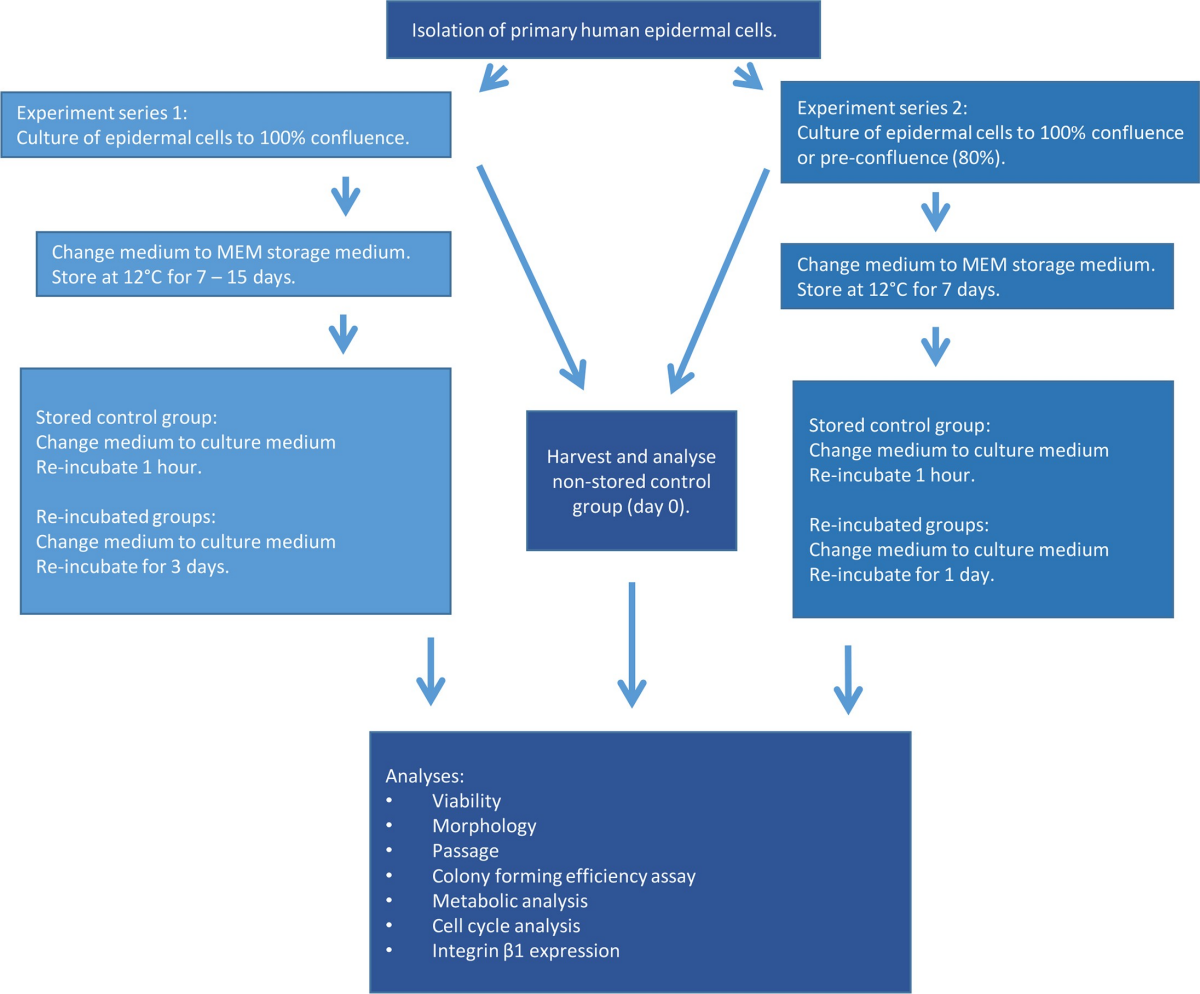
Chart illustrating workflow of the two experimental series and the analyses performed.

The cells were expanded and passaged at 80% confluence. Culture medium was changed every two days. Epidermal keratinocytes between passage 2 and 3 were used for experiments. Cells were seeded at 15 000 cells/cm^2^ on Nunclon 6-well multi-dishes coated with 1 μg/cm^2^ collagen IV and cultured in serum-free CnT-Prime medium under normal conditions (5% CO_2_ and 37°C). CES were put into storage and the non-stored control was harvested and analysed according to experimental set-up (Fig 1). Two series of storage experiments were performed. The first experimental series stored cells for up to 15 days. The second series stored cells for 7 days. A supplementary experiment was also performed where morphology was observed after 3- and 5-days re-incubation following 20 days of storage (S1 Fig). Another supplementary experiment investigated the effect of storing CES either in MEM or CNT under normal incubator conditions (5% CO_2_ and 37°C) for 15 days.

The aim of the 7-day experimental series was to compare storage of confluent and pre-confluent cell sheets. Confluence was assessed visually by phase-contrast microscopy. When cells had achieved 80% growth coverage of the total surface area they were considered pre-confluent. The confluent group were cells cultured 12 hours beyond 100% confluence (overnight) before storage the following morning. Since confluence is a primary signal for differentiation in epidermal cells they were at a more advanced state of differentiation in order to emphasize any changes compared to undifferentiated pre-confluent cells.

### Cell storage

Cells were stored using the protocol previously described [8]. Briefly, following culture, the CNT-Prime culture medium was exchanged for storage medium and each 6-well multi-dish was sealed with Nunclon adhesive sheets and stored at 12°C. The storage medium was MEM with HEPES (25 mM), sodium bicarbonate (3.57 mM) and gentamycin (50 *μ*g/ml). No medium changes were performed during storage. Cultured epidermal cells, not subjected to storage, served as non-stored controls (day 0). Following storage, MEM storage medium was replaced with CNT-Prime medium. Using the method outlined previously [8], the non-re-incubated stored groups were analysed after only 1 hour in the incubator (37°C, 5% CO_2_), whereupon normal morphology was regained. For the 15-day storage series, the media was changed to CNT after 15 days of storage and the re-incubated group was left in the incubator for 3 days. For the 7-day storage experiment the media was changed to CNT and the re-incubated group was left in the incubator for 1 day.

### Cell viability

After storage for up to 15 days cells were washed with PBS and trypsinized (n = 6). Cells from 6-well plates were harvested for counting by incubation with 500 μl trypsin-EDTA (0.25% + 0.01% EDTA) for two minutes. Trypsin was deactivated with 500 μl soybean trypsin inhibitor (2mg soybean powder /1ml CNT-Prime medium). This was done twice to harvest all cells. After centrifugation and resuspension in 1 mL CNT-Prime medium, the cell suspension was diluted 1:10 and counted with a Reichert hemocytometer. Cells infused with trypan blue were deemed non-viable and excluded from cell viability counts.

### Flow cytometry

Analysis of cell cycle, apoptosis, cell size and integrin β1 expression was carried out by flow cytometry after storage for up to 15 days (n = 6). Cells were analysed with a BD Accuri C6 flow cytometer and data were analysed using BD Accuri C6 Software (version 1.0.264.21) and FlowJo software (FlowJo LLC, Ashland, USA).

### Cell cycle and apoptosis analyses

Cells were fixed with 70% ethanol for 30 minutes at 4°C. After resuspension in PBS DNA was stained with propidium iodide (PI; 5μg/mL) for 30 minutes in the dark. Fluorescence data generated by PI staining were collected using the FL2 channel following gates eliminating debris and identifying single cells.

### Cell size and integrin β1 expression analyses

Cells were blocked in staining buffer (PBS, 0.5% BSA, 2mM EDTA) at 4°C for 15 minutes and incubated with FITC-conjugated integrin β1 monoclonal antibody for 30 minutes (2 μl antibody solution per 125 μl staining buffer; mouse anti human CD29-FITC, Bio-Rad. Final IgG concentration 1.57 nanograms/μl). Fluorescence data were collected in the FL1 channel after gating to eliminate debris and identifying single cells.

### Metabolic analysis

After storage for up to 15 days cell samples of storage media from each group were analyzed for glucose, lactate and pH levels storage using a Radiometer ABL 700 blood gas machine (Bronshoj, Denmark) (n = 6).

### Morphology

Phase contrast light microscopy images were taken before and after storage at 3 random positions within each well at 100x, 200x and 400x with Leica DM IL LED microscope and Canon EOS 5D mark II camera (Canon, Oslo, Norway). Counts of cells with vacuoles and apoptotic morphology were performed by visual inspection of two representative photographs from three biological replicates from each group. Cell counts were assessed by two independent researchers.

### Colony Forming Efficiency (CFE) assay

Fibroblast feeder layers were prepared for the CFE assay by culturing NIH/3T3 mouse fibroblasts (ATCC, Manassas VA, USA) in T175 culture flasks with DMEM low glucose medium with 10% FBS and 1% penicillin-streptomycin. They were inactivated with incubation in mitomycin C (4 μg/ml medium) for 4 hours to stop proliferation. Inactivated mouse fibroblasts were trypsinized and seeded in 6-well multi-dishes (45 000 cells/cm^2^) and left to attach for at least 4 hours.

After storage for 7 days 500 epidermal keratinocytes from each experimental group were seeded in a 6-well plate (n = 3) with a fibroblast feeder layer in enriched culture medium (1:1 DMEM/F12 nutrient mix with fetal bovine serum (10%), penicillin-streptomycin (1%), insulin (5μg/mL), hydrocortisone (0.4μg/mL), epidermal growth factor (10 ng/mL), cholera toxin (0.1 nM), adenine (24 μg/mL) and T3 (1.4 ng/mL). The thin seeding ensured that the cells were too far apart to influence each other, giving stem cells an opportunity to form large colonies (holoclones) while non-stem cells formed small, irregular colonies with aborted cell growth or perished [13]. The plates were cultured for 12 days before they were fixed with methanol and stained with crystal violet. Large, regular shaped colonies containing over 50 cells were counted as holoclones, generally accepted as originating from and representing a single stem cell [14]. Small colonies with irregular shape were regarded as originating from transient amplifying cells and not included in colony counts. Percent CFE was calculated by dividing the number of holoclones by the total number of cells seeded (500) and multiplying by 100.

### Passage

Confluent and pre-confluent cells were stored for 7 days (n = 3). The stored cells were compared with cells that had been stored and re-incubated to test proliferation during passaging (n = 3 each group). Cells were counted and seeded at 15 000 cells/cm^2^ in new 6-well plates coated with collagen IV. Morphology was assessed each day.

### Statistical analysis

One-way ANOVA with multiple comparisons between groups and Tukey’s post-hoc analysis was performed in Graphpad Prism 7 for viability, integrin β1, cell cycle analysis and CFE. Analysis of correlation between viability and lactate used Pearson’s correlation.

## Results

### Viability of the cell sheet deteriorates by storage day 11

**Fig 2:**
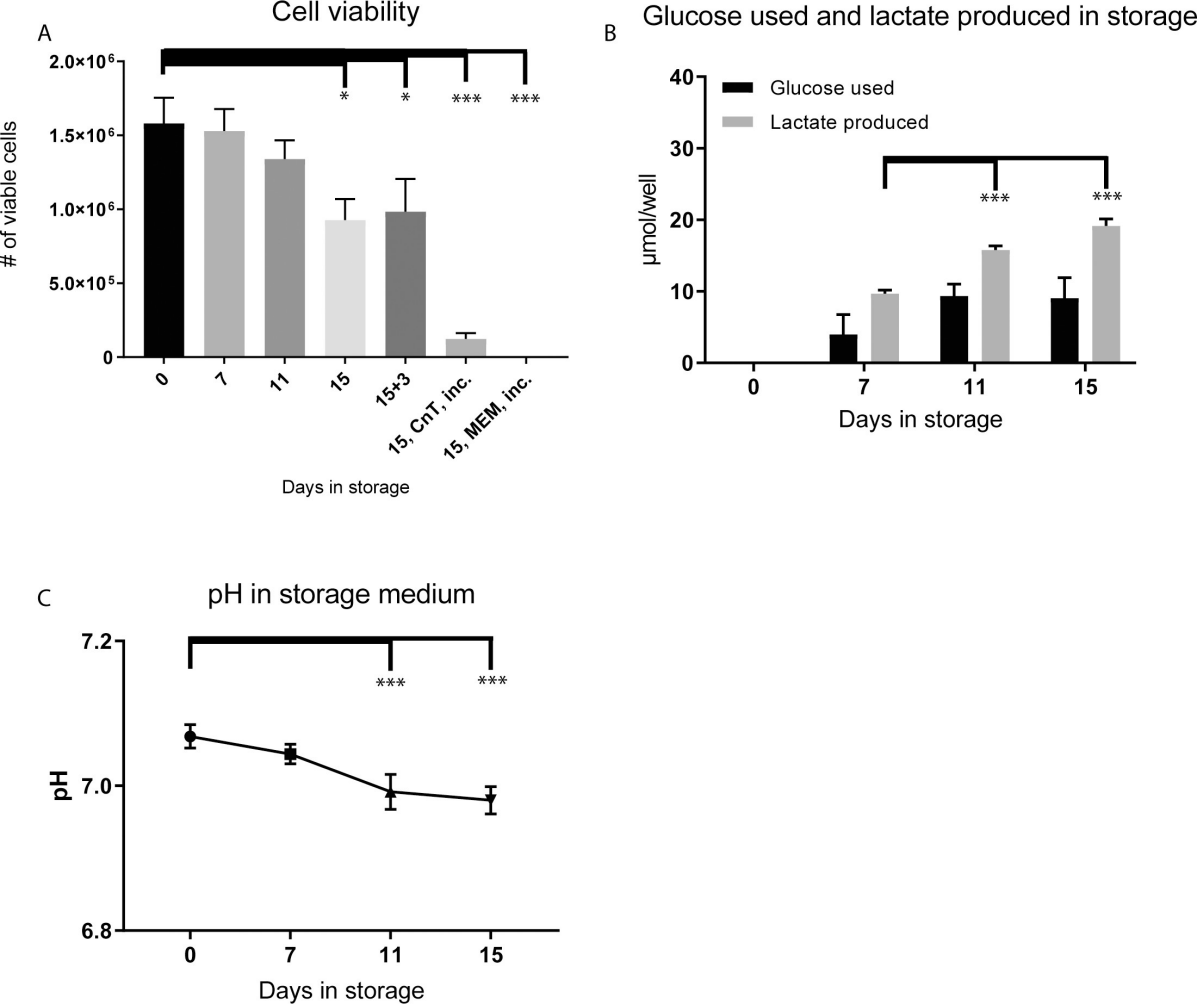
Cell viability and metabolic analyses during 15 days storage and 3 days re-incubation. **(A)** Cell viability showing counts of trypan blue negative cells. Viability significantly decreased between day 11 and 15 in storage. For comparison, cells left in a normal incubator (5% CO_2_, 37°C) throughout the complete 15-day storage period in normal CnT-Prime or MEM storage medium resulted in poor cell survival (n = 6). (**B)** The metabolites glucose and lactate were measured in the storage media before and after storage. The lactate produced was significantly increased at 11 and 15 days compared to 7 days. (**C)** The pH of the storage medium was measured before and after storage. * = significant difference; p≤0.05; *** = significant difference; p≤0.0001.

Cell viability was measured by counting trypan blue negative cells. The cell count in the non-stored control was 1.58±0.17x10^6^ cells. There was a decreasing trend from day 11 (~85% of non-stored control) and the cell count was significantly decreased to 0.93±0.14x10^6^cells by day 15 (~60% of non-stored control; p<0.0001 day 15 vs control; p = 0.0004 day 15 vs day 11: Fig 2A). Three days re-incubation of CES following 15 days storage resulted in no change in the number of viable cells (0.98±0.22x10^6^, ~65% of non-stored control). In comparison, storage of CES in the incubator throughout the complete 15-day storage period in normal CnT-Prime or MEM storage medium (without media exchange) resulted in poor cell survival. The CnT-Prime group had 0.123±0.038x10^6^ cells compared to 1.58±0.17x10^6^ cells in the non-stored control (Fig 2A). All the cells in the MEM storage group had detached and aggregated in the medium and were impossible to count.

Analysis of the metabolites glucose and lactate in the storage medium gave an indication of the level of metabolic activity and the dominant metabolic pathway in use during storage. Increased lactate suggests increased reliance on glycolysis to produce ATP rather than use of the tri-carboxylic acid (TCA) cycle. Lactate significantly increased from 0 μmoles in the non-stored control group to 9.68±0.49 μmoles (p<0.0001) at day 7, 15.77±0.57 μmoles (p<0.0001) at day 11 and 19.14±0.98 μmoles at day 15 (p<0.0001: Fig 2B). Lactate accumulation at 11 and 15 days was significantly increased compared to day 7 (p<0.0001). The amount of glucose used per well was 3.99±2.73 μmoles at day 7, 9.35±1.65 μmoles at day 11 and was similar at day 15 (p = 0.99). Lactate production was strongly negatively correlated with viability (R = -0.837: Fig 2B). The pH of the storage medium at 7 days remained stable with 7.07±0.02 in non-stored control compared to 7.04±0.01 at 7 days (p = 0.11: Fig 2C). The pH significantly decreased compared to non-stored control at day 11 and 15 with 6.99±0.02 (p<0.0001) and 6.98±0.02 (p<0.0001), respectively.

In summary, these results show that viability begins to deteriorate by day 11 of storage and is strongly associated with increased lactate in the storage medium as well as reduced pH. Glycolysis appeared to be the preferred metabolic pathway during storage.

### Cell cycle stage is maintained during 15 days storage

**Fig 3:**
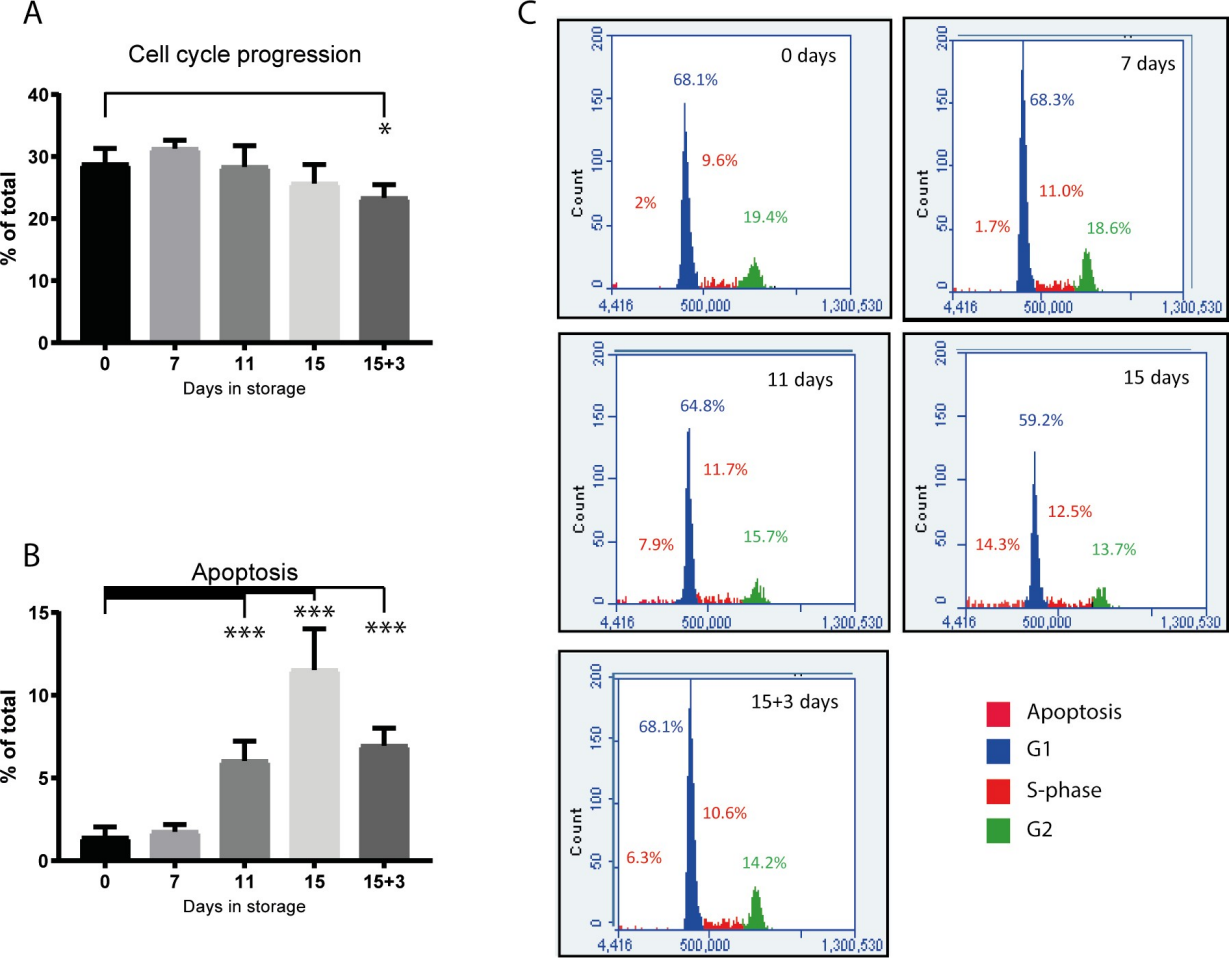
Flow cytometry with Propidium Iodide (PI) was used to measure the percentage of cells preparing to enter or in mitosis (S-, G2- and M-phase combined). The graphs **(A)** and **(B)** were prepared using this flow cytometry data. Representative flow cytometry histograms are shown in **(C). (A)** Number of cells preparing to enter mitosis (S-phase + G2/M) analysed using PI and flow cytometry (n = 6). **(B)** Percentage of apoptotic cells analysed using PI and flow cytometry (n = 6) **(C)** Histograms from representative samples showing distribution of cells in apoptosis, G1, S-phase and G2/M. * = significant difference; p = 0.0287; *** = significant difference; p≤0.0001.

The percentage of cells preparing to enter or in mitosis (S-, G2- and M-phase combined) was measured by flow-cytometry. 28.8±2.5% of cells in the non-stored control compared to 31.2±1.4% after 7 days of storage, 28.4±3.4% at day 11 and 25.7±3.0% at day 15 were in this active phase (changes not significant: Fig 3A & 3C). However, this number dropped to 23.4±2.1% after 3 days re-incubation following 15 days storage (p = 0.0287 compared to control).

The percentage of apoptotic cells in the non-stored control group was 1.4±0.6 (Fig 3B & 3C) with no change after 7 days storage (p = 0.99). A significant increase in apoptosis was seen by day 11 with 6.0±1.1%, increasing to 11.5±2.6% by day 15 (p<0.0001 compared to all groups). After 3 days re-incubation the percentage of apoptotic cells decreased to 7.0±1.1% (p<0.0001). In this case the apoptotic cells accumulated during 15 days storage were likely removed during the change back to CNT-Prime medium for re-incubation.

Combined analyses of cell cycle and apoptosis suggested that 7 days is the optimal storage period for retention of proliferating cells. Highest apoptosis was seen at 15 days storage.

### Cell sheet morphology is disrupted during extended storage but improves with re-incubation

**Fig 4:**
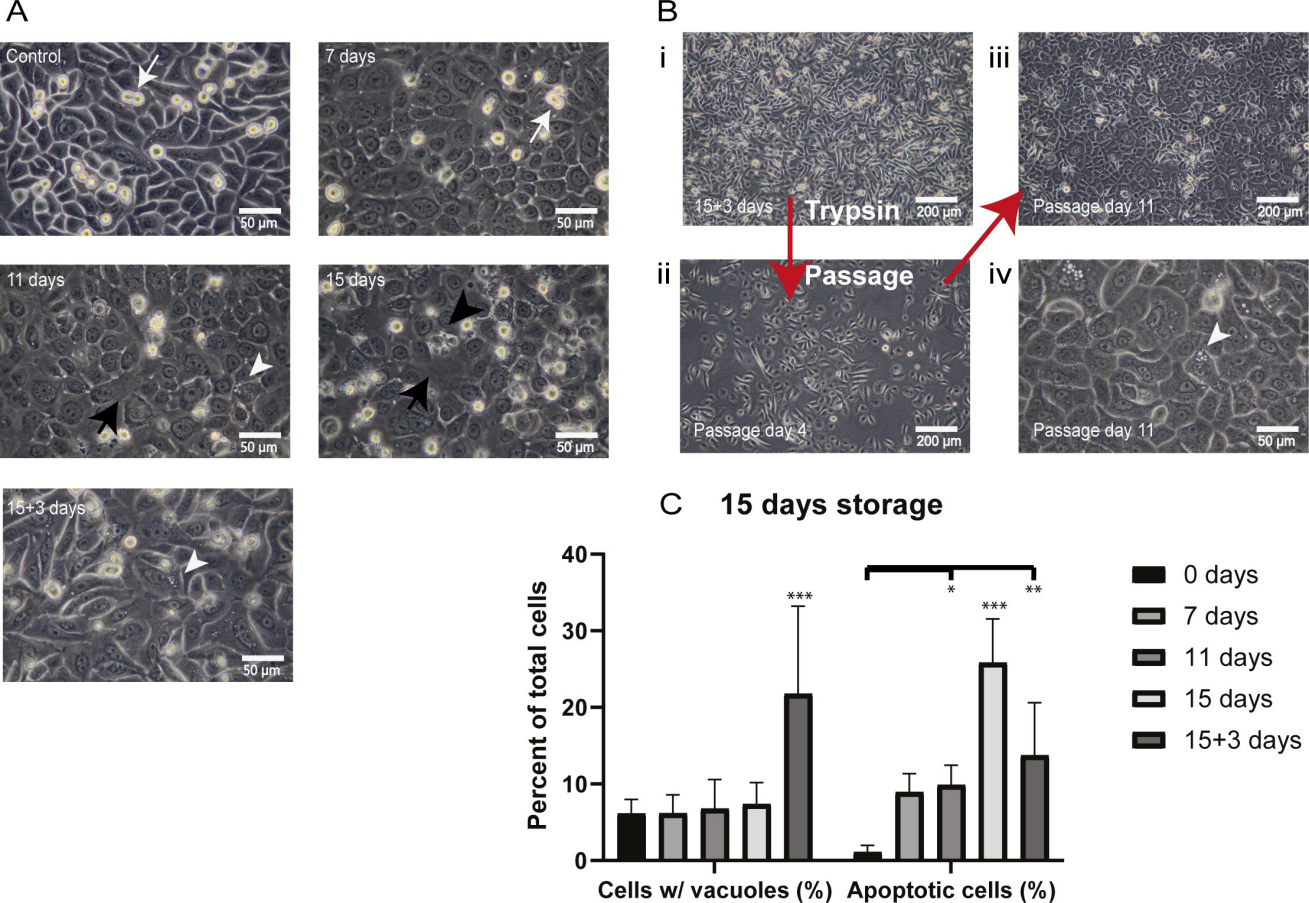
Morphological analysis by phase contrast light microscopy. **(A)** CES stored for 7 days, 11 days, 15 days and 15 days + 3 days re-incubation (magnification: 400X in all groups; n = 6). **(B)** Cells from CES stored 15 days and re-incubated 3 days **(i)** may be trypsinized and seeded to a new plate **(ii),** and grow to become confluent and differentiate by passage day 11 (magnification: **(iii)** 100X and **(iv)** 400X). *White arrows*: mitotic cells. *White arrowhead*: vacuoles. *Black arrows*: gap in cell layer. *Black arrowhead*: detaching/apoptotic cell. **(C)** The graph shows the percentage of apoptotic cells and stressed cells containing vacuoles. Cells were characterized and quantified using 400X magnification photographs (n = 6). *** = Significant difference against all other groups (p<0.0001) ** = Significant difference (p<0.001) * = Significant difference (p<0.05).

Observation of morphology by phase contrast light microscopy showed a mosaic pattern of small and large cells typical of confluent CES in the non-stored control. Dividing cells were also present (white arrows: Fig 4A). Confluence was maintained following 7 days storage and cells with apoptotic morphology were rare. By 11 days of storage gaps in the cell layer were visible (black arrows: Fig 4A) as well as intracellular vacuoles, indicative of cell stress (white arrowhead). After 15 days the changes were more advanced with larger gaps (black arrow) and more detaching cells (black arrowhead). Remarkably, after 3 days re-incubation recovery (5% CO_2_, 37°C) following 15 days storage (15+3 days) the morphology of the cell sheet greatly improved with increased confluence, smaller gaps visible in the cell sheet and fewer detaching cells (Fig 4A). The cells were capable of forming a confluent cell layer after trypsinization and seeding to a new plate (Fig 4B). However, larger cells and the presence of vacuoles were indicative of more differentiated cell morphology and cell stress (white arrowhead). The percentage of cells with vacuoles increased from 6.6%±1.8% at 0 days storage to 21.8%±11.4% (p<0.0001) at 15+3 days storage (Fig 4C). Based on photographs, the number of cells with apoptotic morphology increased to 25.8%±5.7% at 15 days storage and decreased to 13.7%±6.8% upon re-incubation for three days (p<0.05). These results showed the significant effect of temperature in de-activating and re-activating cell activity and demonstrated the potential for cells to partially regain normal cell morphology and function when brought back to incubator conditions (5% CO_2_, 37°C) following an extended storage period at 12°C.

In a supplementary experiment we found that re-incubation of CES for 3 or 5 days following extended (20-day) storage resulted in similar improvements in cell morphology as the 15+3 group (S1A Fig). However, cells with vacuoles were present, suggestive of older cells and cell stress (white arrowheads: S1A Fig). Despite major disruption of the cell sheet (S1A Fig; black arrow), approximately 90% confluency was regained following 3 days culture in a normal incubator. 100% confluence was regained with typical mosaic morphology similar to non-stored control after 5 days re-incubation (S1A Fig). Counts made from visual assessment of photographs from the 20-day storage experiment confirmed our observations that the number of cells with vacuoles and apoptotic cells decreased with re-incubation of 3 or 5 days (S1C Fig). In stark contrast, storage of CES in the incubator throughout the complete 15-day storage period in MEM storage medium or normal CnT-Prime medium resulted in complete disruption of cell sheet morphology in both groups (S1B Fig).

Overall, fewer dividing cells were observed in the re-incubated cell sheets compared to control. However, these results show that re-incubation of CES following extended storage improves cell sheet morphology and restores confluency.

### Functional testing of re-incubated CES stored for 15 days showed cells retain proliferative function and differentiate

Cell sheets stored for 15 days were re-incubated for 3 days (Fig 4B(i)) then trypsinized for passaging. Seeded cells showed clear growth at day 4 after passage (Fig 4B(ii)) and reached 100% confluence by day 7 (not shown) indicating a slower proliferation rate compared to confluence on day 3 or 4 typically achieved with fresh cells. By day 11 after seeding the confluent cells were differentiating (Fig 4B(iii) & 4B(iv)). Importantly, this experiment showed that cells stored for 15 days and re-incubated for 3 days retain sufficient proliferative potential to reach confluence after passaging.

### Cells stored for 7 days showed increased expression of stem cell marker integrin β1

**Fig 5:**
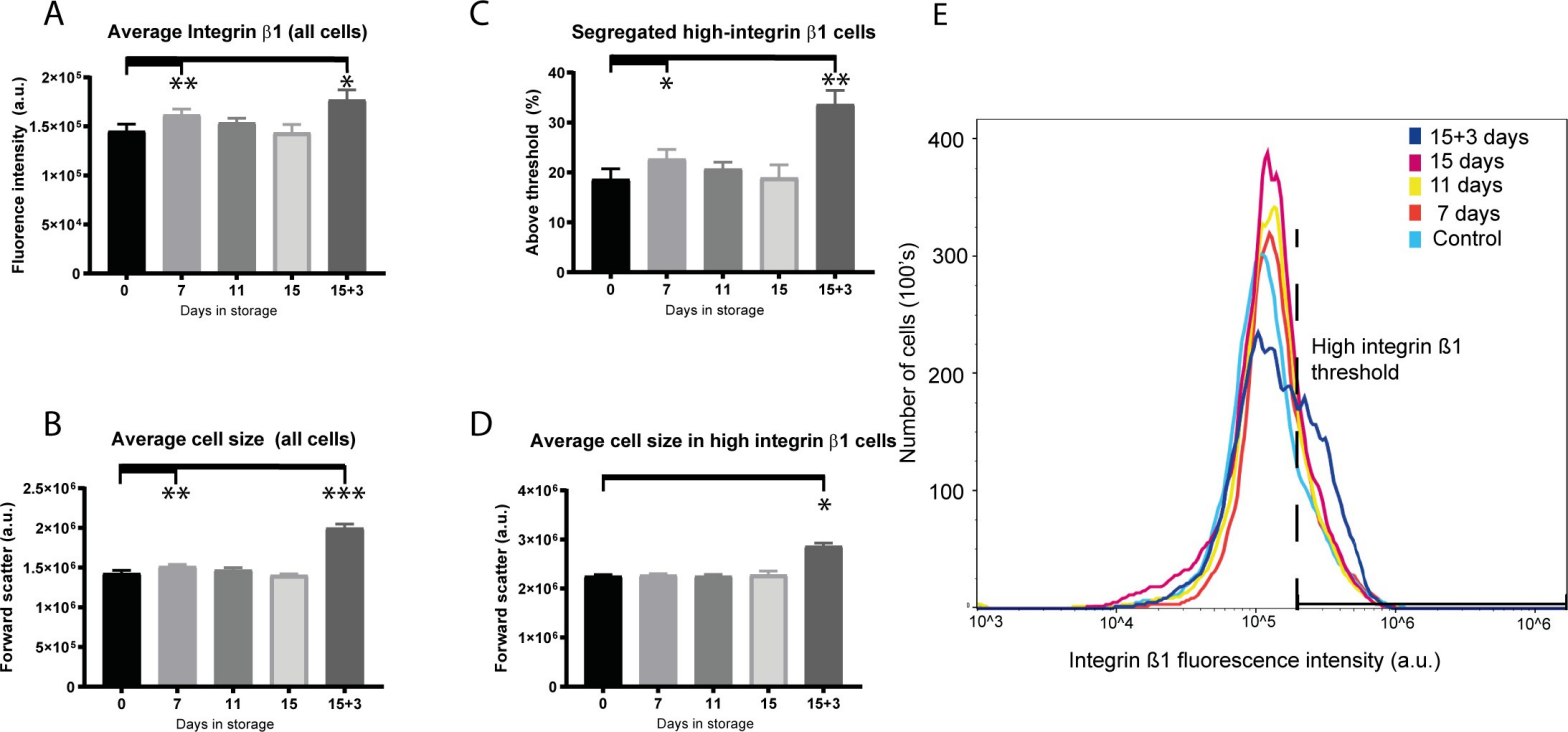
Analyses of cell size and cell membrane stem cell marker integrin β1 expression measured by flow cytometry. **(A)** Average level of integrin β1 expression (n = 6). **(B)** Average cell size as measured by forward scatter (n = 6). **(C)** The percentage of segregated high integrin-β1 expression cells from each group scoring above the 2.0x10^5^a.u. fluorescence threshold. **(D)** Average cell size of the high-integrin β1 expressing cells. **(E)** Plot showing average integrin-β1 expression and threshold of high integrin-β1 expressing cells with fluorescence above 2.0x10^5^a.u. * = significant increase; p≤0.05; ** = significant increase; p = 0.002; *** = significant increase; p≤0.0001.

Integrin β1 expression and cell size were measured by flow cytometry. High expression of membrane adhesion protein integrin β1 combined with small cell size is indicative of stem cell phenotype [15]. Cells stored for 7 days showed higher average integrin β1 expression compared to non-stored control (1.61x10^5^a.u. and 1.45x10^5^a.u. respectively; p = 0.002: Fig 5A). The average expression of integrin β1 was also significantly higher in the 15+3 days re-incubated group with a value of 1.8±1.0 x10^5^a.u. compared to a range of 1.4 to 1.6x10^5^a.u. in all other groups (p = 0.0195: Fig 5A). However, forward scatter was also increased in the 7- and 15+3-days storage groups compared to non-stored control (p = 0.002 and p<0.0001 respectively; Fig 5B) indicating larger cell size.

To further establish whether cells with highest average integrin β1 expression were stem cells that had survived storage better than more differentiated cells or whether high expression was due solely to larger cell size we segregated high-integrin β1 expressing cells by setting a threshold value (2.00x10^5^a.u.: Fig 5C & 5E). We found that ~23% of cells stored for 7 days and ~35% of cells in the 15+3 days re-incubated groups segregated over the high average integrin β1 threshold (Fig 5C & 5E). Cells in the 7 days storage group had similar forward scatter values to the non-stored control (p = 0.953), indicating a higher integrin β1 to cell-size ratio and retention of stem cell phenotype (Fig 5D). Conversely, cells in the 15+3 days re-incubation storage group were significantly larger in size, suggesting that they were likely not stem cells.

Overall, flow cytometry results indicated that cells stored for 15 days and re-incubated for 3 days were larger in size than before re-incubation. CES stored for 7 days contained cells with high average integrin β1 expression suggesting retention of a high percentage of stem cells. Average integrin β1 expression had declined by day 11 of storage.

### Viability and morphology is improved with storage of pre-confluent cells

Pre-confluent cells will typically have superior proliferation compared to confluent cells in normal culture conditions. It is important to transplant cells that have active proliferation capacity upon removal from storage. However, it is unknown how pre-confluence will influence the viability of cells stored at 12°C since confluence provides stability through cell-cell interactions in the cell sheet. For comparison we stored pre-confluent cells at 80% and confluent cells at 12 hours past 100% confluence to emphasize any effects (over-confluent) (n = 3).

**Fig 6:**
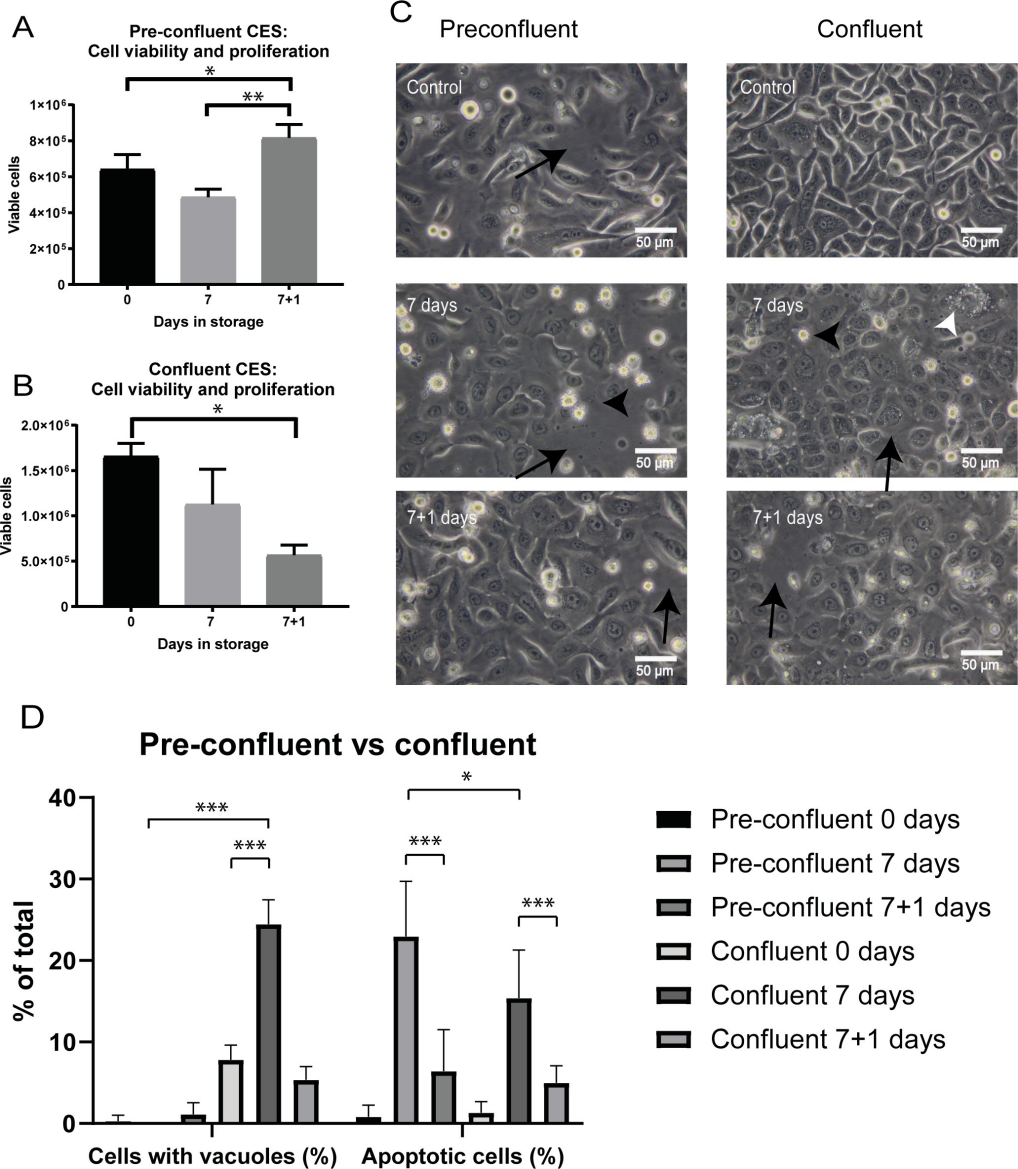
Comparison of the effect of confluence state and 1 day re-incubation on cell viability and cell proliferation **(A)** Cell count of trypan blue negative cells in CES stored at pre-confluence (* = significant increase; p = 0.049; ** = significant increase; p = 0.0028; n = 3). **(B)** Cell count in confluent stored CES (** = significant decrease; p = 0.004; n = 3). **(C)** Morphology of CES, 400X magnification. Black arrows: gap in cell layer. Black arrowhead: detaching/apoptotic cell. White arrowhead: vacuoles. **(D)** The figure shows the percentage of apoptotic cells and stressed cells containing vacuoles. Cells were characterized and quantified using photographs (n = 6). *** = Significant difference (p<0.0001) * = Significant difference (p<0.05).

The non-stored pre-confluent control had a much lower cell count than the over-confluent group due to the shorter culture time. The average number of viable cells in the non-stored pre-confluent control was 0.64±0.08x10^6^ cells (Fig 6A). This number fell to 0.49±0.05x10^6^ cells after storage for 7 days, though this was not significant (p = 0.0773). After recovery of one day re-incubation, the cell number significantly increased to 0.82±0.08x10^6^, exceeding the pre-confluent, non-stored control (p = 0.0499). This represented ~67% increase in viable cells during one day re-incubation following 7 days storage (0.49±0.05x10^6^ cells; p = 0.0028: Fig 6A).

Counts of trypan blue-negative cells showed that the number of viable cells in the over-confluent group tended to decline after 7 days in storage with a count of 1.12±0.39x10^6^ compared to 1.65±0.14x10^6^ cells in the non-stored over-confluent control, though this was not significant (p = 0.0886: Fig 6B). Unlike the pre-confluent group re-incubation for one day did not re-activate cell proliferation in this group. To the contrary, the number of viable cells decreased to 0.56±0.11x10^6^, significantly less than the non-stored over-confluent control (p = 0.0041).

Following 1-day re-incubation the cells in the pre-confluent group had proliferated to form a mosaic pattern (Fig 6C; left column). Confluency increased following re-incubation recovery; there were fewer gaps between the cells (black arrows: Fig 6C; left column) and fewer apoptotic cells visible (black arrowheads) compared to the non-stored control cells analysed immediately after removal from 7 days storage (Fig 6C; left column). Cell morphology closely resembled the freshly cultured cells seen in the non-stored pre-confluent control group. Counts (n = 6) confirmed our initial observations that there were minimal cells with vacuoles either post-storage or post 1-day incubation (Fig 6C; left column & Fig 6D). A count of cells with apoptotic morphology showed that there were significantly more apoptotic cells in the pre-confluent group with 23.0±6.7% compared to the over-confluent group (Fig 6D; p< 0.05). However, the number of cells with apoptotic morphology in the pre-confluent group decreased significantly following 1-day re-incubation and were not significantly higher than the comparable over-confluent group, reflecting viability results (Fig 6A & 6D).

Morphology deteriorated in the over-confluent group. Holes in cell sheets (black arrows: Fig 6C; right column) and cells with apoptotic morphology (black arrowheads) were visible immediately following 7-day storage as well as in the re-incubated group. A count of cells with apoptotic morphology revealed a significant increase to 15.4±5.9% by day 7 (Fig 6D; p<0.0001). Vacuoles were also visible, indicating cell stress (white arrowhead: Fig 6C right column). Moreover, a count of cells with vacuoles showed a significant increase with 24.5±3.0% at day 7 compared to non-stored control over-confluent cells (Fig 6D; p<0.0001).

These results clearly showed that storage at pre-confluence produced far superior results in retention of viable proliferating cells.

### Re-incubation following storage improves stem cell colony formation

The colony forming efficiency (CFE) assay is a functional assay widely used for assessing the number of stem cells in a cell population. In the clinical burns setting it has been used to gauge the likelihood of graft success using cultured autologous keratinocytes from the patient [16]. We assessed the CFE of pre-confluent and over-confluent cells after 7 days storage and following one-day re-incubation recovery. Over-confluent groups were stored at 12 hours post-confluence to emphasize any effects on CFE compared to pre-confluent groups.

**Fig 7:**
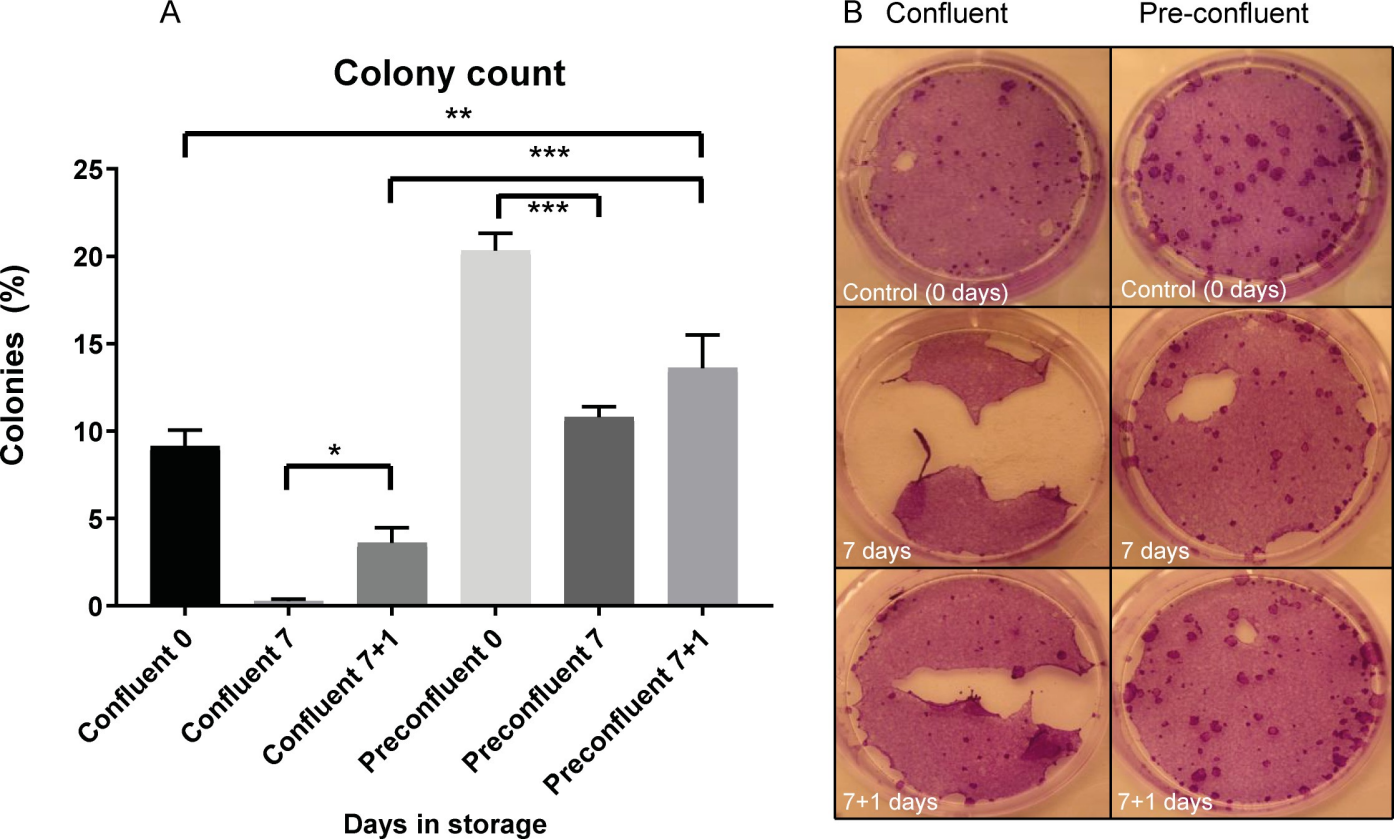
Colony Forming Efficiency (CFE) assay of pre-confluent and confluent cells stored for 7 days with and without one day re-incubation. **(A)** Counts of colonies in pre-confluent groups compared to confluent groups (* = significant increase; p = 0.020; ** = significant increase; p = 0.0023; *** = significant difference; p<0.0001; n = 3). **(B)** Representative images of CFE dishes from each group.

Overall, pre-confluent stored CES had far superior CFE performance compared to the over-confluent stored group (10.60±0.60% vs. 0.26±0.11% at 7 days storage; p<0.0001; Fig 7A & 7B). The pre-confluent group also out-performed the over-confluent group with one day re-incubation (13.60±1.90% vs. 3.60±0.87%; p<0.0001; Fig 7A & 7B).

Before storage, the CFE in the non-stored over-confluent control group was 9.13±0.92% (Fig 7A & 7B). CFE dropped to 0.26±0.11% following 7 days storage. Re-incubating the cells for one day significantly improved CFE to 3.60±0.87% (p = 0.0203;).

The CFE of pre-confluent cells decreased to 10.60±0.60% following storage for 7 days from 20.33±0.98% in non-stored pre-confluent control (p<0.0001 vs. pre-confluent control) but showed a trend to improve with a CFE of 13.60±1.90% after one day of re-incubation (p = 0.058).

In summary, pre-confluent CES had superior retention of stem cells and colony forming ability during storage and re-incubation. These results suggest that use of stored pre-confluent cell sheets would perform best following transplantation.

### Proliferative function is retained in confluent and pre-confluent cell sheets stored for 7 days at 12°C

**Fig 8:**
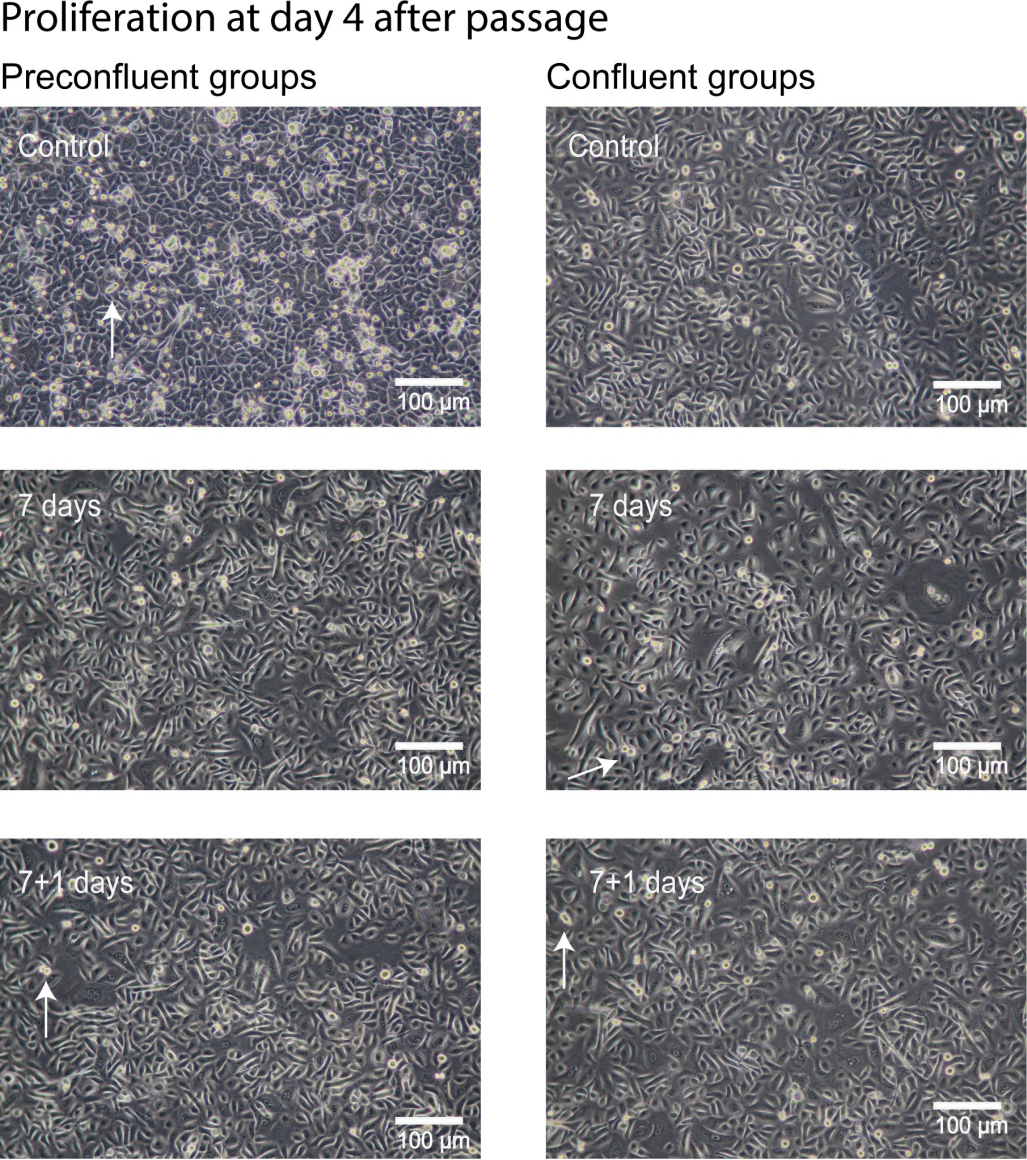
Experimental cells from Fig 5 were passaged. Cells are shown at day 4 after seeding (n = 3), 200X magnification. Cells from all groups could be passaged and grew to confluence. Passaged cells from the pre-confluent groups (left) divided more quickly. *White arrows*: dividing cells.

To further assess the effect of confluence on cell function we tested if cells could be passaged following storage and re-incubation. We found that cell morphology of passaged cells was similar in all groups at day 4 post-seeding (Fig 8). Dividing cells were observed in all groups (white arrows: Fig 8). Confluence was achieved at day 4 post-seeding in the non-stored pre-confluent control group and day 5 in the confluent group (Table 1). The pre-confluent stored cells also reached confluence 1 day earlier than the confluent group (Table 1). No difference between the groups was seen when cells were first incubated for one day before passaging. However, incubation did shorten the time to achieve confluence in the passaged stored confluent group to 5 instead of 6 days indicating that one day of recovery was important to re-activate proliferative function (Table 1).

**Table 1:** Passaged cells: Time to achieve 100% confluence.

Storage Group	Days
Confluent Non-stored control (Day 0)	5
Pre-confluent Non-stored control (Day 0)	4
Day 7 confluent	6
Day 7 pre-confluent	5
Day 7 + 1 day re-incubation confluent	5
Day 7 + 1 day re-incubation pre-confluent	5

The results of passaging experiments showed that proliferative function is retained in both pre-confluent and confluent groups after 7 days storage. Re-incubation improved the proliferation rate of cells harvested and seeded from confluent stored CES sheets.

## Discussion

The storage temperature 12°C was chosen based on studies indicating this is the optimal temperature demonstrated over a storage period of 7–14 days [8, 10, 17]. One of our objectives was to investigate this interval to establish the optimal storage period. Our second focus was to investigate if re-incubating (37°C, 5% CO_2_) CES following storage at 12°C would improve CES proliferation and morphology. Our third main question was to investigate the effect of confluence, especially with regard to retention of stem cell function and proliferative potential of keratinocytes, which is important to post-transplantation success [16].

Maintenance of cell viability and a population of undifferentiated cells are crucial factors for clinical success using CES to treat burns and for their use in regenerative medicine [18, 19]. Our current findings corroborate previous results showing cell viability is maintained when CES is stored at 12°C for 7 days [8, 10]. However, we clarify here that viability begins to decline between the time-points 7 and 11 days and reaches ~60% by day 15 of storage. The increase in apoptosis in CES stored for 11 and 15 days compared to the non-stored control group indicated that cell stress increased significantly beyond day 11, which may limit the clinical usefulness of CES stored for this long. Re-incubation of stored CES resulted in less apoptotic cells, which likely was due to detachment of weakened cells and their removal with media exchange during re-incubation. These flow cytometry results were confirmed by visual inspection and quantification, though counts using flow cytometry were lower, likely due to processing. Overall, results of apoptosis analyses corroborated our viability tests, suggesting that the quality of stored CES starts to deteriorate between 7 and 11 days of storage.

Morphology analysis showed that cell sheet confluence and cell morphology were optimal over 7 days storage, while signs of degradation were observed at 11 and 15 days. This corresponded with analysis of glucose in the storage media, which showed that cells were metabolically active from day 0 to day 11 but then entered a quiescent phase. Lactate accumulation indicated reliance on glycolysis for ATP production. The inverse correlation of lactate with cell viability between storage days 11 and 15 could have been due to release of intracellular lactate following cell death during storage. Conversely, gradual accumulation of lactate in the storage media could have contributed to decreased pH and increased cell death. Cell morphology had severely deteriorated by day 15, with large holes in the cell sheet and detaching cells. In stark contrast, re-incubation of CES for 3 days following 15 days storage resulted in recovery of many of the morphological features of the non-stored control group. However, several factors suggested that the increase in confluency was probably due to increased cell size rather than increased proliferation: the number of viable cells as measured by trypan blue did not increase, the percentage of cells in active proliferation phases of the cell cycle measured by flow cytometry was lower, there were fewer dividing cells and more large cells observed by phase contrast microscopy. This was further supported by the increase in forward scatter measured by flow cytometry, indicating a larger average cell size following re-incubation. Counts revealed that the number of cells with vacuoles also rose sharply after re-incubation suggesting cell stress. These observations were consistent with cell cycle analysis results and suggested cells in the 15+3 group were quiescent and had entered differentiation.

High integrin β1 expression is associated with epidermal stem cells [15]. Stem cells are small and larger cell size is associated with increased differentiation [20]. Segregation of high integrin β1 expressing cells using flow cytometry revealed that average integrin β1 expression was increased in cells stored for 7 days compared to non-stored control, whereas average cell size was unchanged. We therefore speculated that our storage system may provide conditions that selectively enhance retention of stem cells through their stronger attachment and survival compared to transient amplifying or differentiated cells.

Corroborating results of our first series of experiments we found that cell viability and cell sheet morphology were maintained in the confluent CES group at 7 days storage. However, viable cell counts decreased following one day re-incubation. It has been shown in other cell types that re-warming after hypothermia causes cell stress and may induce formation of reactive oxygen species, apoptosis and DNA damage [21], which may account for this decrease. Interestingly, ~25% of the remaining cells in the confluent group had vacuoles following 7 days storage, indicative of cell stress. In contrast, we found that the pre-confluent group had better tolerance to storage and very few cells displayed vacuoles. Though the percentage of cells with apoptotic morphology was increased following storage in the pre-confluent group compared to the confluent group this did not affect the total number of viable cells observed after re-incubation. Moreover, pre-confluent cells responded well to re-incubation becoming highly prolific and increasing in number by ~67%. We hypothesize that the decreased resistance to apoptosis may be due to their lack of stimulus from neighbouring cells. These experiments showed that confluency is an important factor when considering tolerance to the stress of temperature change and pre-confluent cells may be best candidates for transplantation. The contrast in results following one day re-incubation indicated less differentiated pre-confluent cells were superior in retention of proliferative potential and ability to resume normal growth.

We next assessed cell function associated with confluency during storage and re-incubation. A CFE assay was used to assess clonal capacity. Colony formation by stem cells from the pre-confluent group greatly out-performed that of their confluent counterparts following 7 days storage and after one day re-incubation. Nevertheless, re-incubation was found to improve CFE performance of cells taken from both confluency groups. Thus, despite high integrin β1 expression suggesting retention of stem cells after 7 days storage, poor CFE results using cells immediately removed from storage showed that colony-forming function is affected. A period of incubation is necessary to recover colony-forming ability. These results indicated that use of pre-confluent CES and re-incubation for one day following 7 days storage is the optimal combination when considering short-term storage for clinical application.

The cells from stored CES were also passaged as a further test of their functional performance. All groups formed confluent cell sheets with cobblestone morphology typical of cultured epidermal cells. Cells seeded from stored pre-confluent CES formed confluent sheets one day faster than the stored confluent cells. However, re-incubation for one day improved the proliferative rate of confluent CES, shortening the time to confluence by one day. Thus, these results supported our CFE analysis suggesting that use of pre-confluent cells and re-incubation improves functional outcome of CES stored for 7 days at 12°C.

The use of pre-confluent keratinocytes in combination with a membrane or scaffold such as fibrin for treatment of burns patients was suggested in an editorial by Harris *et al*. in 1998 [22]. The CES in our study were all grown on collagen IV coated plastic. Based on the results, an investigation of CES cultured on a fibrin scaffold followed by 7 days storage and one day re-incubation could be an avenue for further study. A biological or artificial scaffold could further improve growth potential of stored CES.

Based on previous work we used the storage temperature of 12°C [8–10]. The current study establishes 7 days as the maximum storage period and shows the added benefit of using pre-confluent cells and employing a recovery re-incubation period following storage. Future efforts to develop a protocol for short-term storage of keratinocytes at above freezing temperatures should consider the recovery period, i.e. sufficient acclimatisation of cells in a normal incubator, before their transplantation. Use of pre-confluent keratinocytes cultured on a suitable scaffold may also be preferable to use of confluent keratinocytes given the higher retention of clonal and proliferative capacity demonstrated in this study. These recommendations may also be applicable to storage of other cell types, such as oral mucosal cells and limbal epithelial cells, which are both in clinical use in regenerative medicine applications.

## Supporting information

S1 Data (XLSX)

S2 Data (XLSX)

S3 Data (XLSX)

S4 Data (XLSX)

S5 Data (XLSX)

S6 Data (XLSX)

S7 Data (XLSX)

S8 Data (XLSX)

S1 Fig Morphological analysis by phase contrast light microscopy. **(A)** CES were stored for 20 days then re-incubated for 3 and 5 days (magnification: 400X). *White arrowhead*: vacuoles. *Black arrows*: gap in cell layer. *Black arrowhead*: detaching/apoptotic cell. **(B)** CES stored for 15 days in the incubator in either a sealed dish with MEM storage medium (left; magnification: 40X) or with CnT-Prime medium that was exchanged every 2 days (right; magnification: 100X). **(C)** The graph shows the percentage of apoptotic cells and stressed cells containing vacuoles. Cells were characterized and quantified using 400X magnification photographs (n = 6). * = (p<0.05); *** = Significant difference against all other groups (p<0.0001).
(DOCX)
