# Supplementary material for: Stem cell function is conserved during short-term storage of cultured epidermal cell sheets at 12°C
Source: PLoS One. 2020 May 20;15(5):e0232270. doi: 10.1371/journal.pone.0232270 (PMC7239464; doi:10.1371/journal.pone.0232270)
Supplement: S1 Fig — (A) CES were stored for 20 days then re-incubated for 3 and 5 days (magnification: 400X). White arrowhead: vacuoles. Black arrows: gap in cell layer. Black arrowhead: detaching/apoptotic cell. (B) CES stored for 15 days in the incubator in either a sealed dish with MEM storage medium (left; magnification: 40X) or with CnT-Prime medium that was exchanged every 2 days (right; magnification: 100X). (C) The graph shows the percentage of apoptotic cells and stressed cells containing vacuoles. Cells were characterized and quantified using 400X magnification photographs (n = 6). * = (p<0.05); *** = Significant difference against all other groups (p<0.0001). (DOCX) [file pone.0232270.s009.docx]

**Supplementary:**

**Fig S1:**


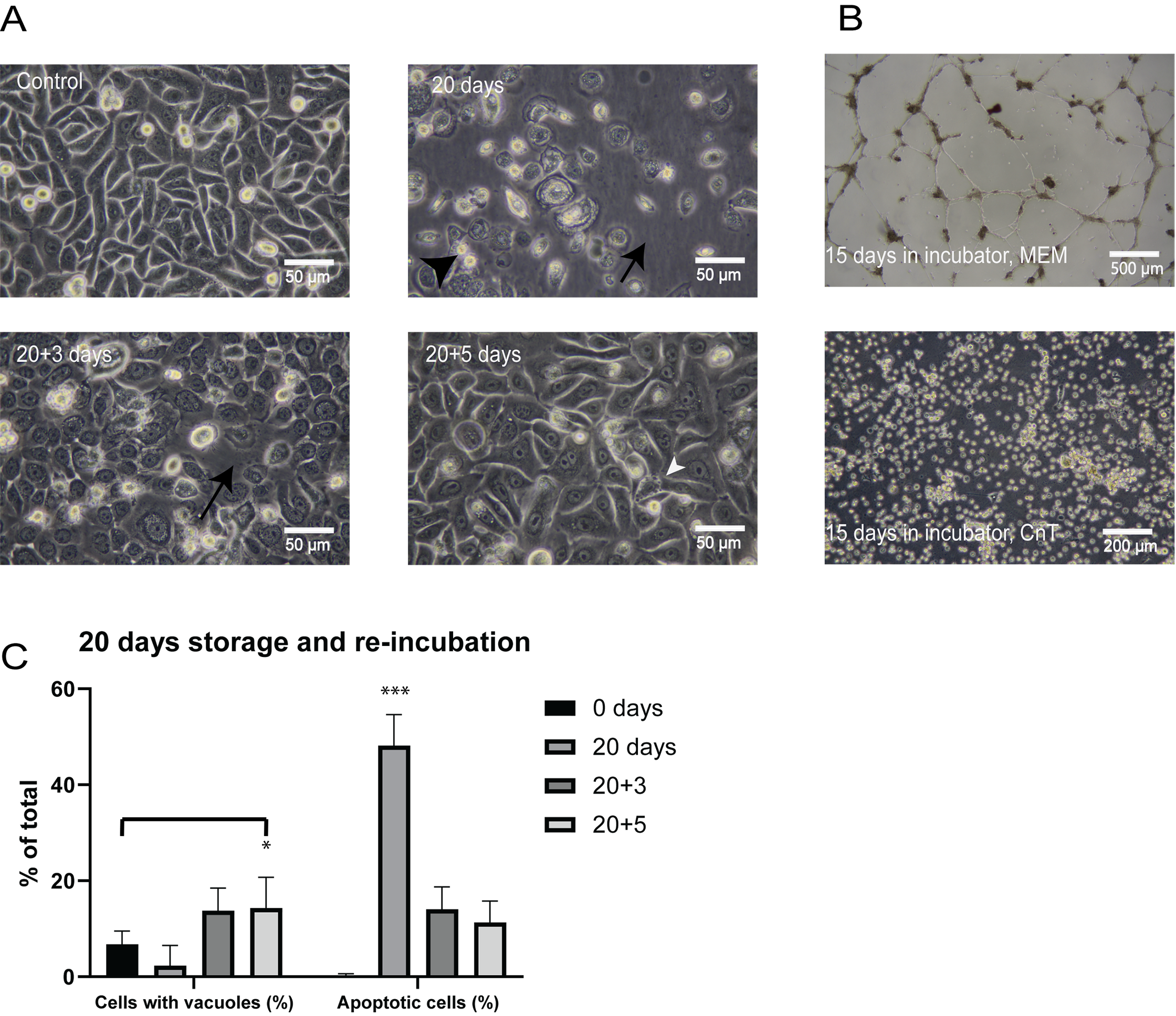


**Fig S1:** **Morphological analysis by phase contrast light microscopy.** **(A)** CES were stored for 20 days then re-incubated for 3 and 5 days (magnification: 400X). *White arrowhead*: vacuoles. *Black arrows*: gap in cell layer. *Black arrowhead*: detaching/apoptotic cell. **(B)** CES stored for 15 days in the incubator in either a sealed dish with MEM storage medium (left; magnification: 40X) or with CnT-Prime medium that was exchanged every 2 days (right; magnification: 100X). **(C)** The graph shows the percentage of apoptotic cells and stressed cells containing vacuoles. Cells were characterized and quantified using 400X magnification photographs (n=6). *= (p<0.05); *** = Significant difference against all other groups (p<0.0001)
